# Lighting a candle in the dark: advances in the genetics and (gene) therapy of retinal dystrophies

**DOI:** 10.1186/1479-5876-10-S3-I3

**Published:** 2012-11-28

**Authors:** Anneke I den Hollander

**Affiliations:** 1 Depts. of Ophthalmology and Human Genetics, Radboud University Nijmegen Medical Centre, Nijmegen, the Netherlands

## 

Retinal dystrophies cause severe visual impairment due to the death of photoreceptor and retinal pigment epithelium cells. These diseases until recently have been considered to be incurable. In the last two decades genetic studies have shed light on the molecular causes of several of these diseases, which has opened new avenues to develop therapeutic approaches. The mammalian eye has been at the forefront of therapeutic trials based on gene augmentation in humans with Leber congenital amaurosis, an early-onset recessive retinal dystrophy, due to mutations in the retinal pigment epithelium-specific protein 65 kDa (*RPE65*) gene. Tremendous challenges still lie ahead to extrapolate these studies to other retinal disease-causing genes, as human gene augmentation studies require testing in animal models for each individual gene and sufficiently large patient cohorts for clinical trials remain to be identified through cost-effective mutation screening protocols.

Pharmacological approaches to antagonize retinal degeneration are also under development, including modulation of the immune system.

